# Takayasu arteritis masquerading as brucellosis: a case report

**DOI:** 10.1093/omcr/omae147

**Published:** 2024-12-10

**Authors:** Karokh F Hama Hussein, Rawa Bapir, Dilan S Hiwa, Nali H Hama, Shorsh A Mohammed, Soran H Tahir, Lawen Jamal Mustafa, Dlshad M Faraj, Hemin S Mohammed, Sokar A Omar, Shvan H Mohammed, Fahmi H Kakamad

**Affiliations:** Scientific Affairs Department, Smart Health Tower, Madam Mitterrand Street, Sulaymaniyah 46001, Kurdistan, Iraq; Gastroenterology Department, Gastroenterology and Hepatology Teaching Hospital, Zanko Street, Sulaymaniyah 46001, Kurdistan, Iraq; Scientific Affairs Department, Smart Health Tower, Madam Mitterrand Street, Sulaymaniyah 46001, Kurdistan, Iraq; Department of Urology, Surgical Teaching Hospital, Zanko Street, Sulaymaniyah 46001, Kurdistan, Iraq; Kscien Organization for Scientific Research (Middle East Office), Hamdi Street, Azadi Mall, Sulaymaniyah 46001, Kurdistan, Iraq; Scientific Affairs Department, Smart Health Tower, Madam Mitterrand Street, Sulaymaniyah 46001, Kurdistan, Iraq; Scientific Affairs Department, Smart Health Tower, Madam Mitterrand Street, Sulaymaniyah 46001, Kurdistan, Iraq; College of Medicine, University of Sulaimani, Madam Mitterrand Street, Sulaymaniyah, Kurdistan, Iraq; Shahid Dr. Hemin Mental Health Hospital, Qanat Street, Sulaymaniyah 46001, Kurdistan, Iraq; Scientific Affairs Department, Smart Health Tower, Madam Mitterrand Street, Sulaymaniyah 46001, Kurdistan, Iraq; College of Medicine, University of Sulaimani, Madam Mitterrand Street, Sulaymaniyah, Kurdistan, Iraq; Rheumatology Department, Ministry of Health, Shorsh Street, Sulaymaniyah 46001, Kurdistan, Iraq; Scientific Department, Xzmat Polyclinic, Rizgari, Kalar, Sulaymaniyah 46001, Kurdistan, Iraq; Scientific Affairs Department, Smart Health Tower, Madam Mitterrand Street, Sulaymaniyah 46001, Kurdistan, Iraq; Scientific Department, Xzmat Polyclinic, Rizgari, Kalar, Sulaymaniyah 46001, Kurdistan, Iraq; Scientific Department, Xzmat Polyclinic, Rizgari, Kalar, Sulaymaniyah 46001, Kurdistan, Iraq; Kscien Organization for Scientific Research (Middle East Office), Hamdi Street, Azadi Mall, Sulaymaniyah 46001, Kurdistan, Iraq; Scientific Department, Xzmat Polyclinic, Rizgari, Kalar, Sulaymaniyah 46001, Kurdistan, Iraq; Scientific Affairs Department, Smart Health Tower, Madam Mitterrand Street, Sulaymaniyah 46001, Kurdistan, Iraq; Kscien Organization for Scientific Research (Middle East Office), Hamdi Street, Azadi Mall, Sulaymaniyah 46001, Kurdistan, Iraq; College of Medicine, University of Sulaimani, Madam Mitterrand Street, Sulaymaniyah, Kurdistan, Iraq

**Keywords:** Takayasu arteritis, pulseless disease, brucella, serology, diagnosis

## Abstract

This study reports a unique case of a 19-year-old male with Takayasu arteritis initially misdiagnosed as brucellosis due to persistently positive brucella serology. Despite multiple anti-brucellosis treatments, symptoms persisted until the correct diagnosis of Takayasu arteritis was made, Subsequent immunosuppressive therapy for Takayasu arteritis led to symptom improvement and normalization of serological markers. This highlights the challenge of distinguishing between these conditions and the potential for immunosuppression to impact brucella serology in such cases.

## Introduction

Takayasu arteritis, frequently called a pulseless disease, constitutes one of the two predominant types of large-vessel vasculitis. Even though its etiology remains unknown, a chronic granulomatous inflammation involving the medium and large arteries, along with their respective branches, results in the aneurysmal degeneration, occlusion, or narrowing of these pivotal vascular structures. It was initially documented among Japanese women and is more prevalent condition in the Asian population, reaching 40 cases per million. The female-to-male ratios consistently span between 5 and 12. It manifests predominantly between 20 and 30 years of age [1–3].

Brucellosis is an infection transmissible between humans and animals, attributable to an intracellular, Gram-negative bacteria belonging to the genus Brucella. This can come about through ingestion of contaminated meat or unpasteurized dairy products, inhalation, or skin contact. It is the most prevalent bacterial zoonosis, surpassing 500 000 reported cases annually [4, 5].

We present the first case report of a Takayasu arteritis patient with a complex and delayed diagnosis due to persistently positive brucella serology and resultant misdiagnosis as brucellosis, with prompt normalization of brucella serology and symptoms after administration of immunosuppressive therapy for Takayasu arteritis. The references have been inspected for credibility based on the most up-to-date criteria [6].

## Case presentation

### Clinical presentation

A 19-year-old male had a history of polyarthralgia, fever, sweating, bouts of severe dry cough, and weight loss over three years. He recently started complaining of bilateral lower limb pain after 10 min of walking, which was relieved by rest. He was a farmer and a non-smoker with no significant medical or surgical history.

### Physical examination

He was slightly pale with normal central pulses and weak peripheral pulses. There was a right subclavian bruit.

### Diagnostic approach

**Figure 1:**
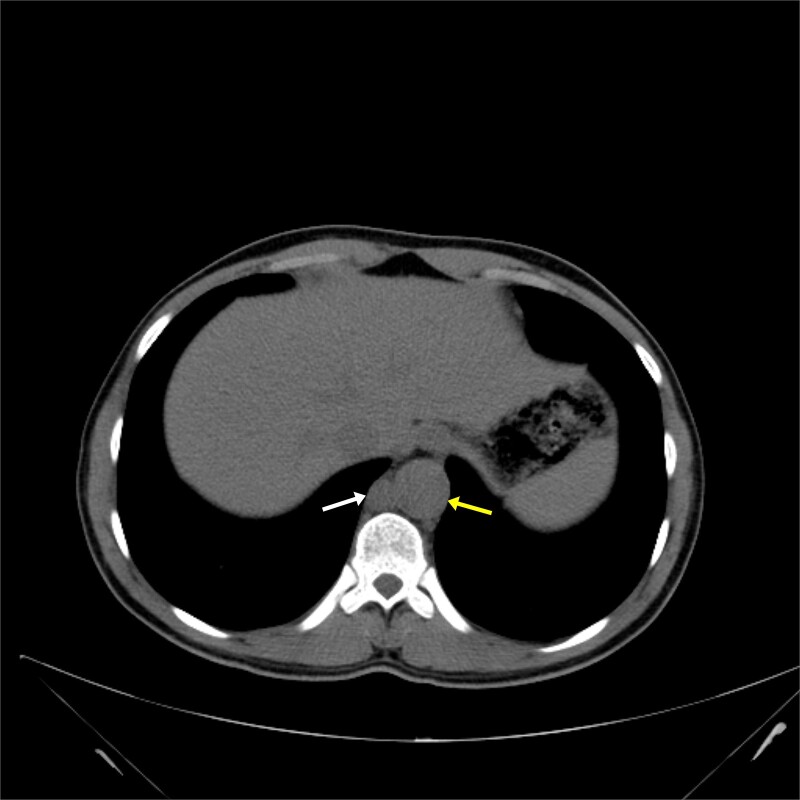
Native chest CT axial section shows mildly dilated thoracic descending aorta measures 3 cm in diameter “yellow arrow,” also inflammatory lymph node 1.1 cm in short axis diameter adjacent to aorta “white arrow”.

The patient’s C-reactive protein (CRP) fluctuated between 35–146 mg/l (reference range < 5 mg/l) for the past two years. Erythrocyte sedimentation rate (ESR) had also fluctuated throughout the past two years between 60–90 mm/h (the reference range for males < 50 years old is 0–15 mm/h). The levels of brucellosis serology were elevated on several occasions which resulted in him receiving five courses of treatment in different medical centers; Brucella IgM and IgG were 13.2 NTU and 16.6 NTU, respectively, (positive reference results > 11.0 NTU, borderline 9–11 NTU). A complete blood count, Anti-nuclear antibodies, anti-Jo1, and Anti-cyclic citrullinated peptides (anti-CCP) were within the normal range. The serology for salmonella IgM and IgG was tested, and the results were negative. The patient has gotten several abdominal ultrasound examinations which all were normal. A high-resolution chest computed tomography (CT) showed several inflammatory lymph nodes around the descending thoracic aorta; the largest was 1.3 cm. Mild dilation of the descending aorta, reaching a size of 3 cm, was also noted (Fig. 1). As the patient had several criteria of Takayasu arteritis (young age, involvement of great vessels, high inflammatory markers), he was suspected to have the disease.

### Treatment and follow-up

The patient was also treated again for brucellosis with a regimen of streptomycin injections (1 gm ×2) for two weeks, doxycycline 100 mg twice daily for six weeks, and rifampicin 300 mg twice daily for six weeks. The ESR, CRP, brucella IgM, and IgG levels were still elevated. After a diagnosis of Takayasu arteritis, he was put on prednisolone 30 mg once daily for two weeks, then tapering gradually on 20 mg and azathioprine 50 mg once daily for three months. His symptoms improved, the inflammatory markers and brucellosis serology were normalized after two weeks of treatment, ESR (6 mm/h), CRP (2.3 mg/l), brucella IgM (8.6 NTU), brucella IgG (8.7 NTU).

## Discussion

An ophthalmologist named Dr. Mikito Takayasou documented the first description of Takayasu arteritis in 1908. Researchers propose that Takayasu arteritis commonly advances through distinct stages, following a triphasic disease pattern. The initial phase, described as the systemic or “pre-pulseless” phase, is marked by constitutional symptoms, which was how the current patient presented. The second phase is characterized by vascular inflammation, which includes notable features such as pain or tenderness specifically localized over arteries. The last phase denotes the vascular damage or “fibrotic” period, marked by limb claudication, stroke, hypertension, or myocardial infarction. The primary criteria utilized for Takayasu arteritis diagnosis is based on the American College of Rheumatology 2022 Classification Criteria. The current patient was less than sixty years old, with reduced peripheral pulses, lower limb claudication, right subclavian bruit, and a thoracic aortic aneurysm on CT scan, fulfilling the diagnostic criteria [2, 7, 8].

The progression of the brucellosis can be delineated into acute, undulant, and chronic forms. Acute brucellosis manifests as an undifferentiated fever, often concomitant with diaphoresis, arthralgias, and malaise. This is why the present case was suspected of having brucellosis. It’s important to highlight that individuals’ immune responses to brucellae vary significantly. In numerous cases where individuals seemingly have entirely recovered from the disease, there may be persisting detectable IgM antibodies for extended periods. Additionally, up to half of patients treated for brucellosis show IgM antibodies even one year after treatment. A swift decline in IgG and IgA antibody titers commonly indicates a favorable response to antibiotic therapy. Conversely, the persistence or escalation of elevated titers may serve as an indicator of treatment inefficacy. Perhaps the patient did get brucellosis, and this might have triggered an immune response for Takayasu arteritis to develop insidiously with their nonspecific symptoms overlapping. Furthermore, the patient received immunosuppressive therapy, which makes brucellosis less likely, as immunosuppressive therapy theoretically should exacerbate infectious diseases. Still, the current case demonstrated clinical improvement and normalization of brucella serology IgG and IgM levels. We propose that the autoimmune and inflammatory interplay of Takayasu arteritis might result in persistently positive brucella serology markers due to an unknown mechanism [4, 5, 9].

## Conclusion

Takayasu arteritis may be misdiagnosed as brucellosis due to persistently positive brucella serology levels and overlapping symptoms in the initial phase of both diseases. In patients not responding to antimicrobial therapy, vasculitis/Takayasu arteritis should be considered in the differential diagnosis. Treating Takayasu arteritis with immunosuppressive therapy may result in the normalization of brucella serological levels.

## Consent

Written informed consent was obtained from the patient for the use and publication of the patient’s medical information in this case report. The next of kin was informed that all efforts would be made to protect the patient’s identity and maintain confidentiality, and consent was provided with full understanding of the publication’s purposes.
